# Targeting glial cell pyroptosis and neuroinflammation in post-stroke depression: from molecular mechanisms to therapeutic strategies

**DOI:** 10.3389/fimmu.2025.1677221

**Published:** 2025-12-11

**Authors:** Xinyao Li, Yuanyuan Wei, Yuqing She, Wenjuan Long, Sitong Zhou, Mingqin Shi, Zihui Wang, Xuelian Zou, Jianqin Mao, Xiangdian Xiao, Hongling Shi, Dongdong Qin

**Affiliations:** 1 School of Basic Medical Sciences, Yunnan University of Chinese Medicine, Kunming, Yunnan, China; 2 First Clinical Medical College, Yunnan University of Chinese Medicine, Kunming, Yunnan, China; 3 College of Traditional Chinese Medicine, Yunnan University of Chinese Medicine, Kunming, Yunnan, China; 4 Second Clinical Medical College, Yunnan University of Chinese Medicine, Kunming, Yunnan, China; 5 Yunnan Key Laboratory of Dai and Yi Medicines, Yunnan University of Chinese Medicine, Kunming, Yunnan, China; 6 School of Traditional Chinese Medicine, Qujing University of Medicine & Health Sciences, Qujing, Yunnan, China; 7 Department of Rehabilitation Medicine, The Third People’s Hospital of Yunnan Province, Kunming, Yunnan, China

**Keywords:** post-stroke depression, glial cell pyroptosis, neuroinflammation, molecular mechanisms, therapeutic strategies

## Abstract

Post-stroke depression (PSD) represents a prevalent and debilitating sequela following cerebrovascular accidents, with its underlying pathophysiology intricately linked to neuroinflammatory processes. Emerging evidence implicates glial cell pyroptosis depending on Caspase-gasdermin D (Casp-GSDMD), orchestrated by the NLR family pyrin domain containing 3 (NLRP3) inflammasome-mediated inflammatory cascades, as a central mechanism in PSD pathogenesis. This review provides a comprehensive analysis of the molecular mechanisms governing glial cell pyroptosis and its dual role in PSD. Specifically, ischemia and hypoxia induce mitochondrial dysfunction and reactive oxygen species (ROS) accumulation, thereby promoting the release of pro-inflammatory cytokines, including IL-1β and IL-18, via the NLRP3/Caspase-1/GSDMD axis. This subsequently exacerbates neuroinflammation and disrupts the blood-brain barrier (BBB) integrity. Furthermore, aberrant activation of pyroptosis-related molecules can trigger neuronal death and impair synaptic plasticity, directly contributing to depressive symptoms. Consequently, therapeutic interventions targeting key nodes within the pyroptosis pathway, such as NLRP3, Caspase-1/4/11, and GSDMD, hold considerable promise, encompassing small molecule inhibitors, natural compounds, and combination therapies. This review synthesizes the multifaceted mechanisms of glial cell pyroptosis in PSD, highlighting the unique therapeutic potential of targeting the pyroptosis pathway to enhance post-stroke neurorepair and mitigate emotional disturbances. These findings may facilitate the identification of novel therapeutic targets and strategies for the diagnosis and management of PSD.

## 1 Introduction

Stroke, a significant contributor to global mortality, usually precipitates PSD, which is a frequently observed sequela in this patient population. Stroke survivors often exhibit pronounced alterations in emotional and behavioral domains (1–3), and the clinical presentation is characterized by a constellation of symptoms, including sustained dysphoria, cognitive deficits, and somatic manifestations (4, 5). PSD incidence has demonstrated a progressive escalation, with reported prevalence rates spanning from 11% to 41% within the two-year period following stroke (6, 7). PSD profoundly affects cognitive function and quality of life, contributing to increased mortality and suicide rates, thereby placing a substantial burden on both societal and familial structures (5, 8). Current first-line pharmacological interventions targeting monoaminergic systems, particularly selective serotonin reuptake inhibitors, demonstrate limited clinical efficacy primarily due to BBB permeability constraints and off-target effects (9, 10). This stimulates the demands for mechanism-based therapeutic advancements.

Emerging evidence implicates neuroinflammation as a critical role in the pathogenesis of PSD (11). Glial cells, encompassing astrocytes, microglia, oligodendrocytes, and oligodendrocyte precursor cells, exhibit a multifaceted role in modulating the inflammatory milieu. Upon activation, these cells secrete pro-inflammatory cytokines, including IL-1β and TNF-α, thereby contributing to neuronal injury (12). Conversely, they may exert an inhibitory effect on excessive inflammation through the regulation of pyroptosis (13). Pyroptosis, a form of programmed cell death, is initiated by inflammasome activation leading to Caspase-1/4 activation and subsequent GSDMD cleavage, culminating in membrane pore formation and the release of pro-inflammatory cytokines (14). Notably, targeting pyroptosis may exert a protective effect by modulating inflammatory responses.

This review will comprehensively analyze the molecular mechanisms underlying glial cell pyroptosis in PSD, specifically focusing on the NLRP3/Caspase-1/GSDMD signaling cascade. We will delineate the pathways by which the release of pro-inflammatory cytokines, including IL-1β and IL-18, compromise the BBB integrity and subsequently trigger neuronal death. In parallel, this review will utilize a dual-target pharmacological approach to investigate pyroptosis-modulating interventions, with a specific emphasis on MCC950-mediated NLRP3 inflammasome suppression and Mavunil-induced GSDMD pore-forming blockade, systematically assessing their neuroprotective capabilities. Finally, we propose a multifaceted therapeutic strategy integrating targeting pyroptosis with immunomodulation. This will provide a deeper understanding of the pathogenesis of PSD, and a theoretical framework for precision interventions in PSD.

## 2 Pyroptosis

### 2.1 Fundamental principles of pyroptosis

Pyroptosis is a caspase-dependent cellular demolition mechanism distinguished by proinflammatory responses. This regulated cell death pathway is primarily mediated by NLRP3 inflammasome activation, which initiates the enzymatic activity of Caspase-1/4/11 effectors to execute the lytic process. Initially characterized in the 1980s, this cell death modality was identified as Caspase-1-dependent, occurring in macrophages stimulated by toxins or infected by pathogens. It is distinguished by Caspase-1-dependent cell swelling and rupture, accompanied by the release of inflammatory mediators such as IL-1β and IL-18 (15, 16). Distinct from apoptosis, pyroptosis is Caspase-3-independent and exhibits robust proinflammatory characteristics, marked by cellular swelling, membrane rupture, and the release of substantial quantities of proinflammatory mediators, including IL-1β and IL-18. This process has been extensively investigated in the context of neurodegenerative disorders (17). Ischemia/hypoxia-induced mitochondrial ROS triggers the NLRP3/Caspase-1/GSDMD pathway, leading to BBB injury and neuroinflammation via cytokine storms (18). Conversely, regulated pyroptosis may facilitate the elimination of compromised glial cells, thereby mitigating inflammatory responses (19). Therapeutic modulation of pyroptosis necessitates a precise calibration of its pro-inflammatory and neuroprotective roles to identify an optimal therapeutic window. Considering pyroptosis’s involvement in neuroinflammatory processes, the canonical and non-canonical inflammasome signaling pathways constitute a critical approach for preventing the development of PSD.

### 2.2 Canonical pyroptosis pathway

Pharmacological targeting of glial cells pyroptotic signaling represents a critical therapeutic strategy to mitigate stroke-induced neuroinflammatory responses and depression-related phenotypes in PSD (20). The NLRP3 inflammasome assembles and enzymatically activates Caspase-1, thereby driving neuronal injury through two primary mechanisms: processing pro-inflammatory cytokine precursors and executing GSDMD-mediated pyroptosis. This protease proteolytically converts pro-IL-1β and pro-IL-18 into their bioactive forms, initiating microglial M1 polarization and concurrently triggering TNF-α and IL-6 secretion (21). This process further amplifies inflammatory signals and directly activates microglia, thereby exacerbating neuronal damage (22). Preclinical investigations have indicated that modulation of the canonical pyroptosis pathway elicits substantial amelioration of the pathological sequelae associated with PSD. Specifically, attenuation of NLRP3 activation diminishes the ensuing pyroptotic cellular response, consequently mitigating the manifestation of depressive-like behaviors (23). Current evidence converges to indicate that pharmacological targeting of the NLRP3 inflammasome-Casp-1-GSDMD axis constitutes a viable strategy for attenuating neuroinflammatory cascades in the pathogenesis of PSD. Emerging evidence indicates that Caspase-4/5/11-mediated non-canonical pyroptosis independently promotes neuroinflammation in post-stroke dementia and may synergize with canonical inflammasome activation, suggesting the need for multi-targeted therapeutic strategies.

### 2.3 Non-canonical pyroptosis pathway

The non-canonical pyroptotic cascade is primarily mediated by Caspase-4/5/11, independent of NLRP3 inflammasome activation. This mechanism is initiated through direct engagement with pathogen-associated molecular patterns, such as lipopolysaccharide (LPS) (24). In PSD, disruption of the BBB may facilitate the infiltration of peripheral LPS into the central nervous system, subsequently activating Caspase-4/11 within glial cells. This activation subsequently cleaves GSDMD to generate GSDMD-N, which forms pores in the cell membrane, thereby triggering pyroptosis (25, 26). Distinct from the canonical pathway, the maturation of IL-1β and IL-18 in the non-canonical pathway remains dependent on Caspase-1, while GSDMD cleavage occurs independently of the NLRP3 inflammasome (27, 28). In PSD models, Caspase-11 inhibition concurrently reduces GSDMD cleavage and NLRP3 activation, suggesting that targeting the non-canonical pathway may synergistically block inflammatory cascade reactions (29). These mechanisms suggest that the non-canonical pyroptosis pathway not only autonomously mediates neuroinflammatory responses in PSD but also potentiates the pathological cascade by augmenting the canonical inflammasome activation, thereby supporting the development of multi-targeted therapeutic interventions.

## 3 Glial cell pyroptosis in PSD

The pathophysiology of PSD is multifaceted, encompassing alterations in neurotransmitter systems, neuroinflammation, neural network disruption, and impairments in synaptic plasticity. Neuroinflammation is recognized as a central mechanism in PSD pathogenesis, significantly influencing the repair processes following secondary brain injury and stroke (30). Moreover, inflammatory mediators may contribute to neuronal injury. IL-1β facilitates M1 polarization of microglia via activation of the NF-ΚB/MAPK pathway, concurrently inhibiting BDNF (brain-derived neurotrophic factor)-TrkB signaling, thereby disrupting hippocampal synaptic plasticity (31). TNF-α directly induces neuronal death through the upregulation of glutamate excitotoxicity and the activation of neuronal apoptotic pathways (32). The GSDMD-N fragment, released during pyroptosis, compromises the integrity of the BBB’s endothelial cells, thereby promoting the infiltration of peripheral immune cells and establishing a positive feedback loop of neuroinflammation (33). These findings demonstrate that glial cell pyroptosis directly promotes depressive-like behaviors by amplifying neuroinflammation, disrupting synaptic plasticity, and compromising BBB integrity. The regulatory threshold of pyroptosis in PSD and its interaction with apoptosis and autophagy pathways remain to be clarified. Given pyroptosis’s key role in neuroinflammation and synaptic dysfunction in PSD, identifying upstream molecular triggers and pathway crosstalk is essential for developing targeted therapies.

### 3.1 Microglial pyroptosis in PSD

Microglia, the intrinsic immune surveillance cells of the central nervous system, serve as the principal mediators of neuroinflammatory processes in PSD. Post-ischemic stimulation induces microglial polarization towards a pro-inflammatory M1 phenotype. M1 microglia release pro-inflammatory cytokines such as IL-1β, IL-18, and TNF-α, and prominently activate the NLRP3 inflammasome/Caspase-1/GSDMD–dependent pyroptotic signaling cascade, thereby exacerbating neuroinflammation and promoting depressive-like behavioral phenotypes (11, 34). The purinergic receptor P2X4 (P2X4R) modulates neuroinflammatory responses by intensifying acute-phase inflammation and facilitating neurotrophic factor support during neural recovery via BDNF secretion, thereby contributing to tissue regeneration and amelioration of depressive symptoms (35). IL-18, a pivotal pro-inflammatory cytokine, engages its specific IL-18 receptor along with the Na-K-2Cl cotransporter 1 to facilitate depression-related phenotypes. Pharmacological inhibition of Na-K-2Cl cotransporter 1 with bumetanide demonstrates promising neurotherapeutic efficacy (36). Pharmacologically, Pharmacologically, DL-3-n-Butylphthalide mitigates PSD by suppressing the TLR4/NF-ΚB signaling pathway, thereby decreasing M1 microglial phenotypic markers and suppressing pyroptosis-associated mediators, such as Caspase-1 and GSDMD (37). Furthermore, the cysteinyl leukotriene 2 receptor antagonist HM3379 mediates antidepressant efficacy through targeted suppression of NLRP3 inflammasome activation and the pyroptosis-mediated cell death pathway (38). Epigenetic modulation also plays a role, as the downregulation of microRNA-34b-3p in hippocampal neurons triggers microglial activation, resulting in neuroinflammatory responses and PSD. This highlights the miR-34b-3p/Eukaryotic Translation Initiation Factor 4E signaling pathway as a promising therapeutic target (39). Critically, microglia do not function independently, and they engage in significant bidirectional communication with astrocytes (40, 41). Microglia orchestrate astrocytic responses through diffusible signaling pathways, which then influence microglial polarization. This intricate glial network critically determines the neuroinflammatory progression in PSD (42, 43).

### 3.2 Astrocytic pyroptosis in PSD

Astrocytes are essential for maintaining synaptic neurotransmission and preserving the integrity of the blood-brain barrier integrity, and their degeneration is a key pathological hallmark in PSD. The NLRP3/Caspase-1/GSDMD mediated pyroptosis is a major contributor to this neurodegeneration. Remarkably, pyroptotic cell death accounts for 56.7% of astrocyte apoptosis in the hippocampus, and pharmacological blockade of this pathway markedly mitigates depressive phenotypes in the PSD murine mode (44). Mechanistically, the K-ATP channel subunit directly interacts with NLRP3 within astrocytes, functioning as a negative regulator of inflammasome assembly. Selective astrocytic ablation of Kir6.1 enhances stress-induced pyroptosis and depressive phenotypes, with these effects attenuated by the administration of the NLRP3 inflammasome inhibitor VX-765 (45). In addition to mediating intrinsic neuronal apoptosis, astrocytes play a crucial role in intercellular communication through the secretion of extracellular vesicles. Extracellular vesicles secreted by astrocytes carrying miR-29a modulate pyroptotic pathways and neuroinflammatory responses via mechanisms involving the tumor protein p53-inducible nuclear protein 1, NF-ΚB, and NLRP3 inflammasome inhibition, providing a promising therapeutic strategy for ischemic cerebral injury (46). Further research supports that targeting the NLRP3/Caspase-1/GSDMD inflammasome signaling pathway or related microRNAs such as miRNA-27a presents an effective therapeutic approach for attenuating astrocytic pyroptosis and the development of PSD (47). It is now established that astrocytes function not only as passive glial scaffolding but also as active components in the immune surveillance mechanisms of the central nervous system (CNS) (48). Their substantial impact on synaptic architecture and neurotransmission highlights their pivotal role in the neuropathological processes underlying PSD pathogenesis (49).

### 3.3 PSD-induced glial cell pyroptosis

**Figure 1:**
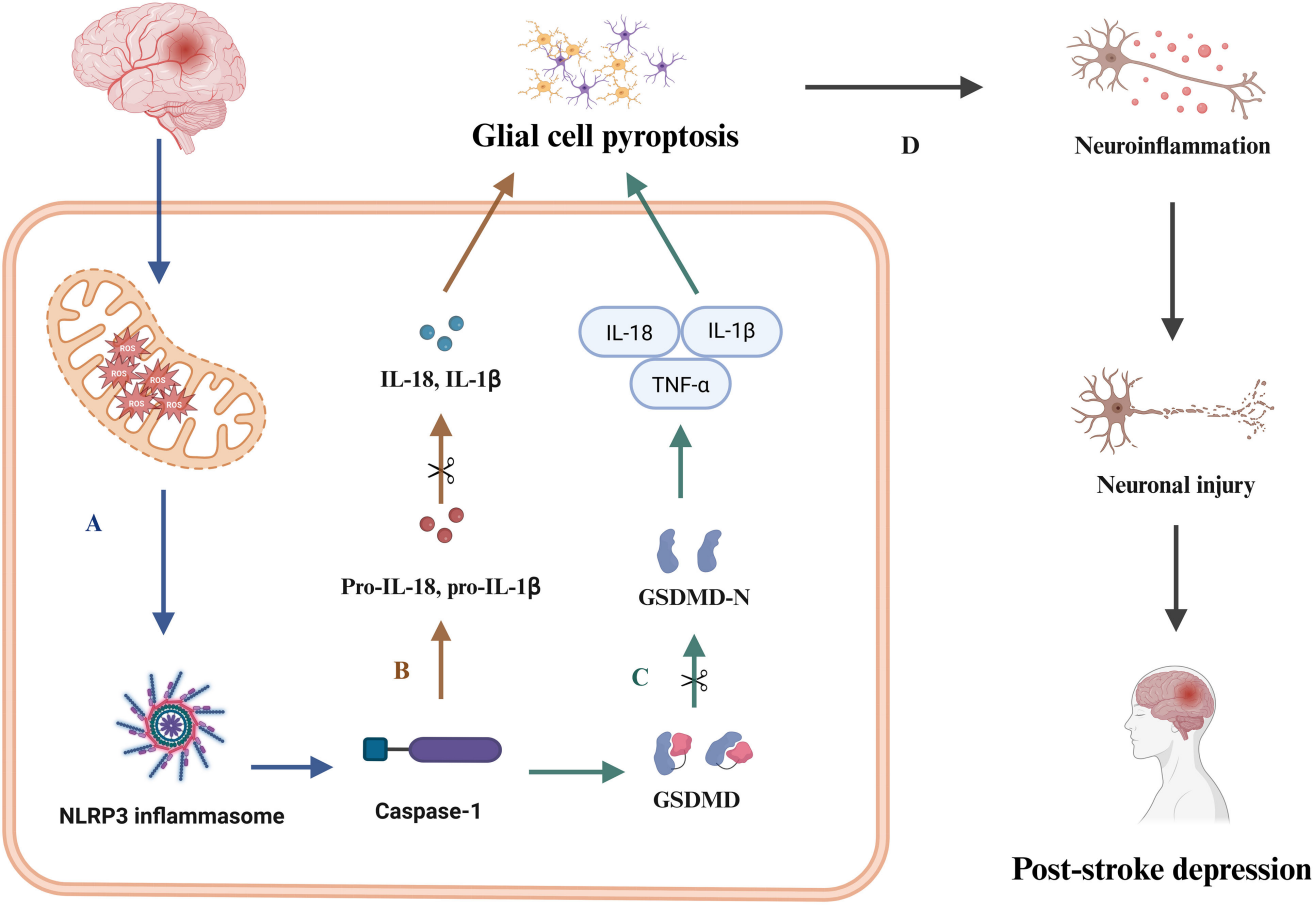
Neuroinflammation-excitotoxicity-mitochondrial dysfunction in PSD. This figure illustrates the molecular signaling cascade within glial cells following ischemic stroke. **(A)** Cerebral ischemia and hypoxia induce mitochondrial dysfunction and accumulation of ROS, with ROS serving as critical mediators for the assembly of the NLRP3 inflammasome and subsequent Caspase-1 activation. **(B)** Active Caspase-1 cleaves pro-inflammatory cytokine precursors pro-IL-1β and pro-IL-18 into their bioactive forms, which lead to glial cell pyroptosis. **(C)** Concurrently, active Caspase-1 cleaves GSDMD to produce the GSDMD-N, which oligomerizes to form membrane pores, facilitating the release of pro-inflammatory cytokines through the GSDMD-N channels, such as IL-1β, IL-18, and TNF-α, which further aggravate glial cell pyroptosis. **(D)** The intensified glial cell pyroptosis perpetuate a neuroinflammatory cycle that exacerbates neuronal injury and contributes to the pathogenesis of PSD. PSD, post-stroke depression; IL-18, interleukin-18; IL-1β, interleukin-1 beta; TNF-α, tumor necrosis factor-alpha; ROS, reactive oxygen species; NLRP3, NOD-like receptor family pyrin domain containing 3; GSDMD, Gasdermin D; GSDMD-N, Gasdermin D N-terminal fragment.

Glial cell pyroptosis is a central mechanism driving the amplification of the PSD neuroinflammatory cascade and subsequent neuronal damage. Activated via the NLRP3/Caspase-1/GSDMD axis, it releases key pro-inflammatory cytokines, thereby dramatically amplifying neuroinflammation. Furthermore, it induces widespread neuronal damage through direct activation of neuronal death programs and indirect pathways, establishing a self-perpetuating vicious cycle of neuroinflammation-excitotoxicity-mitochondrial dysfunction, as shown in **Figure 1**.

Stroke-induced glial cell pyroptosis contributes to the development of depression. Ischemia and hypoxia initiate glial cell ferroptosis. Post-stroke cerebral hypoperfusion induces mitochondrial dysfunction, impairing the electron transport chain and leading to ROS accumulation (50). Mitochondrial damage releases damage-associated molecular patterns such as mtDNA, which activate the TLR4/NF-ΚB pathway to induce NLRP3 transcription (51). Inflammasome activation is promoted by ROS and mtDNA, which facilitate NLRP3-NEK7 interaction, upregulate NLRP3 expression, and recruit ASC and pro-Caspase-1 to form the inflammasome complex (52). Activated Caspase-1 cleaves GSDMD to form membrane pores, inducing cell swelling and lysis (53). Caspase-1 cleaves pro-IL-1β and pro-IL-18, releasing active cytokines (54).

The resultant inflammatory cytokines, such as IL-1β, compromise the BBB, thereby augmenting brain injury (55) Moreover, inflammatory cytokines released during pyroptosis, including IL-1β, IL-18, and TNF-α, activate Caspase-1 within neurons, initiating apoptotic and pyroptotic cell death (56). Trans-cinnamaldehyde exerts neuroprotective effects by reducing the levels of pro-inflammatory cytokines such as IL-1β, decreasing the overexpression of synaptic proteins, and inhibiting the activation of the TLR4/MVD88/MAPKS pathway (57). Simultaneously, IL-1β disrupts hippocampal synaptogenesis by inhibiting BDNF-TrkB/PSD-95/Synapsin-1 signaling (58), thereby establishing a self-perpetuating cycle of excitotoxicity and mitochondrial dysfunction (59). Furthermore, these inflammatory mediators may indirectly induce neuronal injury by activating N-methyl-D-aspartate (NMDA) receptors, resulting in excessive glutamate release and excitotoxicity (60). TNF-α upregulates NMDA receptor activity, resulting in excessive glutamate release, calcium overload, and mitochondrial membrane potential collapse. This ultimately activates the Bax/Bcl-2 apoptotic pathway, leading to the collapse of neuronal mitochondrial membrane potential and neuronal death (61).

Hippocampal-dependent cognitive impairment is modulated by circuits involved in emotional integration, cognitive regulation, and emotional output (62). Impairment of synaptic plasticity and neuronal loss directly disrupt the prefrontal cortex-hippocampus-amygdala circuit involved in emotional regulation (63), thereby involving the emergence of depressive-like behaviors. In animal models, NLRP3 inflammasome inhibition reduced hippocampal inflammation and PSD-associated pyroptosis, indicating NLRP3 as a potential therapeutic target for PSD (64). Guarana oligosaccharides suppress NLRP3 inflammasome activation in microglia, reducing hippocampal inflammation and alleviating PSD (65). Stroke-induced ischemia and hypoxia activate glial cell pyroptosis, releasing inflammatory mediators that cause synaptic injury and neuronal apoptosis, disrupting emotional circuits and contributing to PSD pathogenesis.

### 3.4 NLRP3/Caspase-1/GSDMD axis

Glial cell pyroptosis amplifies post-stroke neuroinflammation via the NLRP3/Caspase-1/GSDMD axis. Post-stroke ischemia and hypoxia induce mitochondrial dysfunction, resulting in elevated ROS production and mtDNA release. These DAMPs stimulate NLRP3 transcription and assembly through the TLR4/NF-ΚB pathway (51). Subsequently, JNK1/GSDMD-mediated NEK7 phosphorylation promotes NLRP3 inflammasome assembly (66). GSDMD-N-mediated membrane perforations facilitate the extracellular release of IL-1β/IL-18, resulting in the spillage of cytoplasmic contents and the propagation of paracrine activation cascades within adjacent glial and neuronal populations (67). IL-1β initiates NF-ΚB/MAPK pathway activation via ligand-receptor engagement, promoting microglial M1 polarization and astrocytic pro-inflammatory activation while suppressing BDNF-TrkB signaling, ultimately compromising hippocampal synaptic plasticity (68). Simultaneously, IL-18 enhances glutamate excitotoxicity through IL-18R/NKCC1 signaling, correlating with depressive phenotypes (36). These observations highlight the therapeutic potential of multi-target interventions against NLRP3 inflammasome activation, GSDMD pore formation, and cytokine networks to mitigate the neuroinflammatory cascade in PSD.

## 4 Therapeutic mechanisms targeting glial pyroptosis

Current PSD interventions are limited by BBB permeability, single-target specificity, and systematic adverse effects, thereby failing to effectively modulate the neuroinflammation-pyroptosis-neuronal injury cascade. Emerging therapeutic strategies targeting glial pyroptosis exhibit pleiotropic effects. Small molecule inhibitors, such as NLRP3/Caspase-1 antagonists, attenuate inflammasome activation, whereas GSDMD inhibitors mitigate pore-mediated neurotoxicity. Natural compounds synergistically suppress pyroptotic pathways and promote neurorestorative signaling. Combinatorial approaches, incorporating nano-delivery systems and epigenetic modulation, enhance spatiotemporal precision. While, psychological interventions such as cognitive behavioral therapy and rehabilitation training can partially ameliorate depressive symptomatology, as they fail to address the fundamental pathological mechanisms of neuroinflammation in PSD, exhibiting limited efficacy in severe cases (69). Consequently, the exploration of novel therapeutic targets and strategies has become a central focus of contemporary research. Among the therapeutic strategies for PSD, targeting glial pyroptosis may represent a novel and potentially efficacious approach. Therefore, targeting the core molecular pathways of glial pyroptosis, include the NLRP3 inflammasome, Caspase family, and GSDMD, offers promise in overcoming the therapeutic limitations of PSD through precise modulation of neuroinflammation and cell death, as detailed in **Table 1**.

**Table 1:** Pharmacological modulation of neuroinflammatory cascades in experimental paradigms relevant to PSD and their underlying mechanisms.

Experimental model	Intervention	Target	Effects and mechanism	References
Chronic unpredictable mild stress (CUMS) mouse model	MCC950	NLRP3 Inflammasome	Improves depressive symptoms by inhibiting the NLRP3 inflammasome, reducing the number of microglia, shifting their activation state to a resting state, and decreasing the expression of NLRP3 and IL-1β in microglia	(53)
Middle cerebral artery occlusion (MCAO) rat model	Astilbin	NLRP3 Inflammasome	Defends against cerebral ischemia/reperfusion injury by inhibiting the NLRP3 inflammasome, while suppressing the MAPK pathway and activating the PI3K/AKT signaling cascade, thereby mitigating cerebral infarction and neurological deficits	(54)
Clinical patients	Chaihu Shugan San	NLRP3 Inflammasome	Components such as paeoniflorin synergistically inhibit the NLRP3 inflammasome and modulate glial cell polarization	(55)
Photothrombosis-induced focal ischemia mouse model	miR34b-3p and eukaryotic translation initiation factor 4E	NLRP3 Inflammasome	Improves depressive symptomatology via microglial inactivation and neuroinflammation inhibition through eIF4E silencing, thereby reversing hippocampal metabolic deficits induced by miR34b-3p dysregulation	(56)
A transient MCAO mouse model	Vx-765	Caspase-1	Caspase-1 inhibition mitigates neuroinflammation by reducing pro-inflammatory cytokines, increasing anti-inflammatory cytokines, and shifting microglial polarization, thereby resolving inflammation and promoting tissue repair	(57)
A thrombin-activated microglial model and intracerebral hemorrhage (ICH) rat model	Ac-YVAD-cmk	Caspase-1	Through the inhibition of Caspase-1 activation, reduces the release of mature IL-1β/IL-18, attenuates brain edema, and decreases microglial activation and the expression of inflammation-related factors	(58)
Primary cultured astrocytes model	H1 receptor activation	Caspase-1	Reduces antipsychotic-induced astrocyte pyroptosis and inflammation by inhibiting NLRP3 inflammasome activation and suppressing pyroptotic signaling	(59)
Lipopolysaccharide (LPS)-induced mouse model	Meranzin hydrate	Caspase-4	Promotes neuronal activity and synaptic plasticity in the hippocampal region by inhibiting Caspase-4 activation, which preserves glial cell function, stabilizes neuro-glial interactions, and supports synaptic remodeling, thereby enhancing cognitive and neural network resilience	(60)
Primary cultured C57BL/6 mouse astrocytes model	Toll-like receptor 4	Caspase-11	Mitigates METH-induced neuroinflammation by inhibiting the Toll-like receptor 4-Caspase-11 pathway, which suppresses NLRP3 inflammasome activation, reduces pro-inflammatory cytokine production, and blocks NF-ΚB signaling in astrocytes	(61)
Carotid arterial injury model in mice	Ganglioside GA2	Caspase-4/11	Alleviates intimal hyperplasia by blocking GA2-mediated macrophage pyroptosis via inhibiting Caspase-4/11 activation, which suppresses the Caspase-9-Caspase-3 pathway and gasdermin E cleavage	(62)
Mouse models of intracerebral hemorrhage	Didymin	Caspase-1/GSDMD	Alleviates neuroinflammation and brain injury by inhibiting microglial pyroptosis via upregulating RKIP, which disrupts ASC inflammasome assembly and suppresses the Caspase-1/GSDMD pathway	(63)
Mouse model of intracerebral hemorrhage	EGCG	Caspase-1/GSDMD/NLRP3	Reduces neuroinflammation by upregulating heme oxygenase-1 expression, which suppresses the Caspase-1/GSDMD/NLRP3 pathway, inhibits microglial pyroptosis, and decreases NLRP3 inflammasome activation and inflammatory cytokine release	(64)
BV2 microglial cells model	NLRC4 inflammasome	NLRC4/Caspase-1/-8/GSDMD	Mitigates ischemic stroke-induced neuroinflammation and microglial cell death by inhibiting the NLRC4 inflammasome, which suppresses apoptotic and pyroptotic pathways via Caspase-1/-8 blockade	(65)
Oxygen-glucose deprivation/reperfusion cell model and CD73 knockout and overexpression mouse models	CD73	GSDMD	Inhibits GSDMD-mediated astrocyte pyroptosis by activating the A2B/NF-ΚB pathway through catalyzing AMP into adenosine and triggering adenosine receptor signaling, which suppresses pyroptotic pathways and reduces inflammatory cell death in astrocytes	(66)
BV2 and HT2 cells model	Caspase-1/GSDMD	Caspase-1/GSDMD	Alleviates cerebral I/R-induced pyroptosis and cell injury by inhibiting the caspase-1/GSDMD pathway, which reduces pyroptosis in both BV2 and HT22	(67)

### 4.1 Inhibition of the NLRP3 inflammasome

The NLRP3 inflammasome is a central regulator of pyroptosis. Its activation induces Caspase-1-dependent proteolytic cleavage of GSDMD, liberating IL-1β/IL-18, thereby propagating neuroinflammatory cascades. Pharmacological inhibition of the NLRP3 pathway, such as with MCC950, may ameliorate glial pyroptosis by suppressing inflammasome oligomerization. MCC950 administration subsequently attenuated Caspase-1 activation and IL-1β secretion, mitigating hippocampal microglial pyroptosis and depression-related symptoms in rodents (70). The natural compound aspergillic acid inhibits NLRP3 activation by scavenging ROS in both OGD/R (oxygen-glucose deprivation/reoxygenation) and MCAO (middle cerebral artery occlusion) models, thereby alleviating neuronal damage (71). Chaihu Shugan San exerts a therapeutic effect on PSD through various active ingredients, such as saikosaponin, hesperidin, paeoniflorin, total glucosides of paeony, glycyrrhizic acid, and total flavonoids of licorice, which inhibit the NLRP3 inflammasome and promote the polarization of microglia towards the M2 phenotype (72). Notably, epigenetic regulation may influence pyroptosis by modulating upstream signals of NLRP3. In post-stroke hippocampal neurons, epigenetic strategies such as miR-34b-3p targeting eIF4E activate microglia, leading to neuroinflammation and PSD. Inhibiting eIF4E expression in hippocampal neurons can reduce neuroinflammation and improve PSD-like symptoms, providing a novel avenue for gene therapy (39).

### 4.2 Modulation of Caspase-1/4/11 activity

Canonical Caspase-1 and non-canonical Caspase-4/11 are critical mediators of pyroptosis, thereby rendering their enzymatic activity as key therapeutic targets. Pharmacological Caspase-1 inhibition by Vx-765 attenuates IL-1β maturation and GSDMD cleavage, thereby mitigating ischemic injury through modulation of pro- and anti-inflammatory cytokine profiles (73). Likewise, Ac-YVAD-cmk-mediated Caspase-1 blockade diminishes the release of IL-1β/IL-18 and microglial M1 polarization (74). These observations underscore the necessity for comprehensive neuroprotection evaluations of caspase-targeting agents, given the dual roles of pyroptosis pathways in neuroinflammatory exacerbation and repair (75). In the non-canonical pathway, Meranzin hydrate, derived from Fructus Aurantii, protects hippocampal synaptic plasticity via caspase-4 inhibition (76). Meanwhile, toll-like receptor 4 (TLR4) can induce neuroinflammation via the astrocyte Caspase-11 signaling pathway (77), whereas ganglioside GA-2-mediated Caspase-11 drives macrophage pyroptosis (78). Further elucidation of the spatiotemporal regulation of canonical/non-canonical caspase networks is essential for the development of stage-specific PSD therapies that simultaneously promote neuronal survival and circuit restoration.

### 4.3 Blockade of GSDMD-mediated pyroptosis

GSDMD serves as the terminal effector molecule of pyroptosis, where its GSDMD-N forms membrane pores leading to cell lysis. Studies have demonstrated that GSDMD inhibitors exhibit promising therapeutic effects in models of neuroinflammation and cerebral hemorrhage, significantly reducing neuroinflammation and depressive-like symptoms (79). Epigallocatechin-3-gallate pretreatment, a non-canonical strategy, enhances heme oxygenase-1 expression, inhibits the Caspase-1/GSDMD/NLRP3 axis, reduces microglial pyroptosis and neuroinflammation, and promotes M1-to-M2 reprogramming, thus conferring neuroprotection (80). Natural compounds intervene in pyroptosis through multi-target actions. In ischemic stroke, the NLRC4 inflammasome complex is activated in microglia, mediating inflammatory responses, apoptosis, and pyroptosis, leading to neuronal cell death and impaired neural function (81). In cerebral ischemia, CD73-mediated adenosine generation activates adenosine receptors, suppressing GSDMD-driven astroglial pyroptosis via A2B/NF-ΚB signaling, thereby reducing neuroinflammation and cerebral injury (82). *In vitro* experiments have shown that activating the Caspase-1/GSDMD signaling pathway induces pyroptosis in microglia and neurons, exacerbating cell damage, while inhibiting Caspase-1 or GSDMD activity can reduce pyroptosis (83). These studies suggest that targeting GSDMD requires consideration of both its upstream activation and downstream effects.

## 5 Conclusion and prospects

Combined treatment strategies can more effectively inhibit glial pyroptosis and neuroinflammation by targeting multiple molecular pathways, thereby improving the therapeutic effect and reducing side effects. MCC950, for instance, inhibits the NLRP3 inflammasome, leading to the inactivation of the NLRP3/Caspase-1/IL-1β signaling pathway in the prefrontal cortex. This reduction in the number of microglia shifts their activation state to a resting state, thus lowering proinflammatory cytokines and preventing stress-induced neuroinflammation (70). The CMC-EXPL nano-preparation has been shown to significantly enhance antidepressant activity and functional capacity in PSD rats, and also reduce brain inflammation, demonstrating the therapeutic potential for PSD (84). In non-drug therapies, three-needle electroacupuncture has been found to improve depressive-like symptoms in a mouse model of PSD by promoting the formation of excitatory synapses in the NGL-3/L1cam pathway (85). Given the increased permeability of the BBB after PSD, the concentration of miRNAs in the blood can serve as an indicator of stroke prognosis, aid in predicting rehabilitation outcomes, and potentially be used as a novel diagnostic target for central nervous system injuries, offering broad application prospects (86). However, these strategies are still in the research and exploration stage, requiring further scientific validation and clinical trials to evaluate their safety and effectiveness.

This review systematically explored PSD, a common and severe complication following stroke, focusing on its pathophysiology related to neuroinflammation mediated by glial cell pyroptosis. The study untangled the critical role of glial cell pyroptosis in PSD and its therapeutic potential. Research findings revealed that ischemia and hypoxia trigger mitochondrial dysfunction, leading to the release of ROS and mtDNA. The signaling cascade initiates NLRP3 inflammasome activation, leading to Caspase-1 activation and GSDMD processing, thereby forming membrane pores. These pores facilitate the extracellular release of IL-1β and IL-18 through lytic pathways. Therapeutic strategies targeting the pyroptosis pathway include inhibiting the NLRP3 inflammasome, blocking Caspase-1/4/11 activity, interfering with GSDMD pore formation, and utilizing natural compounds and novel nanomaterials.

These approaches confirm that glial cell pyroptosis represents a potential therapeutic target for PSD. However, current therapies face challenges such as limited brain targeting, immunosuppression risks, and individual differences. Future investigations should focus on elucidating the subtype-specific mechanisms of glial cell pyroptosis, developing spatio-temporal dynamic regulation tools, exploring multi-omics mechanisms, and developing nano-delivery systems for clinical translation, as well as investigating individualized therapeutic strategies. Through multi-dimensional optimization, targeting pyroptosis holds promise as a new pathway for precise intervention in PSD and other neuropsychiatric disorders.
